# Small RNA and degradome deep sequencing reveals drought‐and tissue‐specific micrornas and their important roles in drought‐sensitive and drought‐tolerant tomato genotypes

**DOI:** 10.1111/pbi.12533

**Published:** 2016-02-09

**Authors:** Bilgin Candar‐Cakir, Ercan Arican, Baohong Zhang

**Affiliations:** ^1^ Programme of Molecular Biology and Genetics Institute of Science Istanbul University Vezneciler Istanbul Turkey; ^2^ Department of Biology East Carolina University Greenville NC USA; ^3^ Department of Molecular Biology and Genetics Faculty of Science Istanbul University Vezneciler Istanbul Turkey

**Keywords:** deep sequencing, degradome, drought, microRNA, signal transduction, tomato

## Abstract

Drought stress has adverse impacts on plant production and productivity. MicroRNAs (miRNAs) are one class of noncoding RNAs regulating gene expression post‐transcriptionally. In this study, we employed small RNA and degradome sequencing to systematically investigate the tissue‐specific miRNAs responsible to drought stress, which are understudied in tomato. For this purpose, root and upground tissues of two different drought‐responsive tomato genotypes (*Lycopersicon esculentum* as sensitive and *L. esculentum* var. *cerasiforme* as tolerant) were subjected to stress with 5% polyethylene glycol for 7 days. A total of 699 conserved miRNAs belonging to 578 families were determined and 688 miRNAs were significantly differentially expressed between different treatments, tissues and genotypes. Using degradome sequencing, 44 target genes were identified associated with 36 miRNA families. Drought‐related miRNAs and their targets were enriched functionally by Gene Ontology (GO) and Kyoto Encyclopedia of Genes and Genomes (KEGG) pathway analyses. Totally, 53 miRNAs targeted 23 key drought stress‐ and tissue development‐related genes, including DRP (dehydration‐responsive protein), GTs (glycosyltransferases), ERF (ethylene responsive factor), PSII (photosystem II) protein, HD‐ZIP (homeodomain‐leucine zipper), MYB and NAC‐domain transcription factors. miR160, miR165, miR166, miR171, miR398, miR408, miR827, miR9472, miR9476 and miR9552 were the key miRNAs functioning in regulation of these genes and involving in tomato response to drought stress. Additionally, plant hormone signal transduction pathway genes were differentially regulated by miR169, miR172, miR393, miR5641, miR5658 and miR7997 in both tissues of both sensitive and tolerant genotypes. These results provide new insight into the regulatory role of miRNAs in drought response with plant hormone signal transduction and drought‐tolerant tomato breeding.

## Introduction

Drought is one of the destructive environmental stress condition that restricts crop production and reproduction in plants (Ding *et al*., 2013). Plants cope with drought stress by recruiting drought avoidance and/or drought tolerance mechanisms at the morphological, physiological, biochemical, cellular and molecular levels such as decreased stomatal conductance, photosynthesis and respiration alterations, production of antioxidant and scavenger compounds, osmotic re‐adjustment and hormonal metabolism changes (Bartels and Sunkar, 2005; Bhargava and Sawant, 2013; Ding *et al*., 2013; Fang and Xiong, 2015). All of these strategies cause gene expression induction and accumulation of some enzymes and drought‐related proteins (Ding *et al*., 2013; Ramachandra Reddy *et al*., 2004; Shinozaki and Yamaguchi‐Shinozaki, 2007). Control of gene expression is one of the regulatory mechanisms on plant response to drought (Golldack *et al*., 2011). Epigenetic regulations such as methylation, histone modifications and post‐transcriptional alterations are stress‐inducible mechanisms and have important roles in stress tolerance (Bhargava and Sawant, 2013).

In plants, small RNA‐mediated regulatory mechanisms play crucial roles on biological processes including growth, development, maturation, transposon silencing, response to abiotic stress and pathogen defence (Xie *et al*., 2012; Li and Zhang, 2016). Small RNAs, especially microRNAs (miRNAs), regulate abiotic stress‐related gene expression post‐transcriptionally, down‐regulating their target genes, while their expression changes conversely (Carrington and Ambros, 2003; Ding *et al*., 2013; Sunkar, 2010; Zhang, 2015; Zhang and Wang, 2015). Many miRNAs response to drought stress via signal transduction pathways such as auxin signalling, ABA‐mediated regulation, osmoprotectant biosynthesis and scavenging of antioxidants (Ding *et al*., 2013). Drought‐related miRNAs and their targets have been identified in different plants such as *Arabidopsis* (Liu *et al*., 2008; Sunkar and Zhu, 2004), cotton (Xie *et al*., 2015), switchgrass (Xie *et al*., 2014) rice (Zhao *et al*., 2007; Zhou *et al*., 2010) and soya bean (Kulcheski *et al*., 2011). However, no systematical study has been performed on tomato.

Tomato (*Lycopersicon esculentum*) is an economically important crop due to demand for fresh vegetable market and processed food industry worldwide (Klee and Giovannoni, 2011). Tomato contains strong antioxidant enzymes, high level of lycopene, rich iron content, and vitamins A and C (Rai *et al*., 2013). Tomato is also one of the most favoured models for fleshly fruit ripening and epigenetic researches (Gonzalez *et al*., 2013; Klee and Giovannoni, 2011). Tomato growth, productivity and nutritional quality are generally affected by environmental stress factors such as drought, salt, flooding and pathogen infection (Rai *et al*., 2013). Cultivated tomatoes (*L. esculentum*) are known as sensitive to drought at all stages of plant development, while seed germination and early seedling stages are mostly affected (Foolad *et al*., 2003). However, their wild types such as *L. pimpinellifolium*,* L. pennellii*,*L. chilense* and *L. esculentum* var. *cerasiforme* are drought‐tolerant species (Sadashiva *et al*., 2013). Researches are in progress to clarify molecular pathways on drought response of plants (Sadashiva *et al*., 2013), and miRNAs can be alternatives to identify drought metabolism, as they play crucial role on abiotic stress response regulating the stress‐related mRNAs (Kumar, 2014). Determination and functional characterization of stress‐related miRNAs and target identification are important in breeding programmes and can contribute to developing new strategies for improving stress tolerance (Barrera‐Figueroa *et al*., 2013). Although several studies have been performed on tomato miRNAs (Cao *et al*., 2014; Feng *et al*., 2014; Karlova *et al*., 2013; Korir *et al*., 2013; Moxon *et al*., 2008; Pilcher *et al*., 2007), there is no study on genomewide drought‐responsive miRNA identification of tomato in tissue‐specific manner.

In this study, we aimed to identify miRNAs and determine their expressions in different tissues under drought stress. For this purpose, we sequenced both root and upground tissues of drought‐sensitive (*L. esculentum*) and drought‐tolerant (*L. esculentum* var. *cerasiforme*) tomato genotypes. Also we carried out degradome sequencing for identifying the targets of drought‐related miRNAs. From here, we identified a total of 699 conserved miRNAs belonging to 578 families, in which 688 miRNAs were differentially expressed between different treatments, tissues and genotypes. We also identified 44 target genes associated with 36 miRNA families. These miRNAs and their targets play an important role in tomato response to drought stress.

## Results

### Data mining of small RNA sequencing

**Figure 1:**
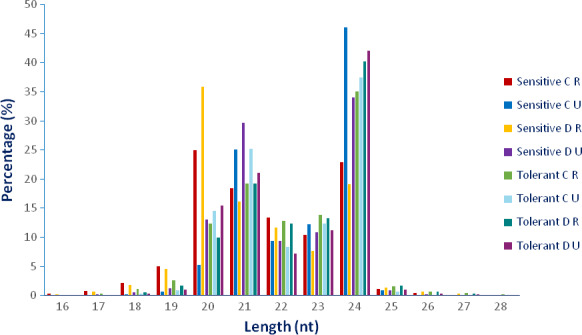
Length distribution of unique small RNAs in drought‐sensitive and drought‐tolerant tomato roots and upgrounds. C, control; D, drought; R, root; U, upground.

To reveal tissue‐specific and tolerance‐related miRNAs under drought conditions, eight small RNA libraries were constructed and sequenced. These samples included four drought‐treated (D) and four untreated (C) samples, in which each contained two roots (R) and two upgrounds (U) tissues belonging to drought‐sensitive *L. esculentum* (S) and drought‐tolerant *L. esculentum* var. *cerasiforme* (T) genotypes. After sequencing, a total of 194 625 986 raw and 192 387 328 clean reads were obtained with the average of 24 328 248 and 24 048 416 (98.85%) reads in each library, respectively (Table S1). The clean tags were used for analysing the distribution of 16‐ to 28‐nt‐length small RNAs and 94.78% of them were determined among 20–24 nt, while the most abundant sRNAs were 21 and 24 nt lengths with the percentage of 21.75% and 35.60%, respectively (Figure 1). However, the small RNA distributions of two libraries (Sensitive C‐R and Sensitive D‐R) were a little different from common results, in which 20‐nt small RNAs are also dominant except 21‐nt and 24‐nt small RNAs. This has not been reported in other plant species. However, the reason for this is unclear; 20‐nt small RNAs may have some role in this situation. About 70.29% and 69.10% unique and 83.72% and 83.01% redundant reads were mapped to tomato genome database (ITAG Release 2.4) in root and upground libraries, respectively (Tables 1 and 2). Using the blastn and blastall alignments against Genbank and Rfam databases, small RNAs were annotated to root and upground tissues of tomato genotypes which have different response to drought stress. In root libraries, most abundant RNA class was rRNA for unique reads with the mean value of 2.32%, followed by tRNA (0.23%), snRNA (0.13%) and snoRNA (0.12%). As for redundant reads, tRNAs were most abundant (21.41%) and other RNAs had quantity with the average of 10.47% (rRNA), 0.23% (snRNA) and 0.17% (snoRNA) (Table 1). For upground libraries, the annotation results were similar and rRNA proportion was highest (1.26%) in unique reads followed by tRNA (0.13%), snRNA (0.06%) and snoRNA (0.04%). In redundant reads, tRNA amount was 10.78% with the highest value, followed by rRNA (3.65%), snRNA (0.05%) and snoRNA (0.03%) (Table 2).

**Table 1:** Small RNA categorization in tomato roots

	Unique (Chavez Montes, #305)	(%)	Redundant (Chavez Montes, #305)	(%)	Unique (S‐D)	(%)	Redundant (S‐D)	(%)	Unique (T‐C)	(%)	Redundant (T‐C)	(%)	Unique (T‐D)	(%)	Redundant (T‐D)	(%)
Mapped	3 494 084	64.49	18 673 483	80.77	2 851 287	64.46	18 884 586	82.60	5 121 311	76.84	19 065 768	85.72	5 941 378	75.35	23 827 315	85.80
rRNA	124 649	2.30	2 780 848	12.03	139 283	3.15	2 446 445	10.70	139 546	2.09	2 269 466	10.20	138 216	1.75	2 485 796	8.95
tRNA	11 811	0.22	5 888 558	25.47	13 324	0.30	8 635 509	37.77	14 586	0.22	2 757 133	12.40	15 134	0.19	2 774 291	9.99
snRNA	7938	0.15	62 136	0.27	7213	0.16	50 819	0.22	7336	0.11	57 384	0.26	7 788	0.10	45 713	0.16
snoRNA	7281	0.13	45 602	0.20	6740	0.15	44 012	0.19	6359	0.10	37 937	0.17	6 244	0.08	34 406	0.12
miRNA	38 135	0.70	1 922 795	8.32	32 852	0.74	1 688 075	7.38	49 802	0.75	2 213 510	9.95	55 393	0.70	2 803 937	10.10
Unann	5 228 253	96.50	12 418 033	53.72	4 223 685	95.49	9 996 894	43.73	6 446 901	96.73	14 906 564	67.02	7 662 721	97.17	19 628 169	70.68
Total	5 418 067		23 117 972		4 423 097		22 861 754		6 664 530		22 241 994		7 885 496		27 772 312	

**Table 2:** Small RNA categorization in tomato upgrounds

	Unique (Chavez Montes, #305)	(%)	Redundant (Chavez Montes, #305)	(%)	Unique (S‐D)	(%)	Redundant (S‐D)	(%)	Unique (T‐C)	(%)	Redundant (T‐C)	(%)	Unique (T‐D)	(%)	Redundant (T‐D)	(%)
Mapped	4 140 172	62.59	17 577 661	76.50	3830 562	62.49	18 565 085	79.66	4 962 483	76.19	21 966 600	88.26	5 101 529	75.13	22 104 275	87.64
rRNA	79 061	1.20	1 093 011	4.76	92 586	1.51	867 283	3.72	70 614	1.08	642 359	2.58	85 932	1.27	896 898	3.56
tRNA	7116	0.11	827 402	3.60	10 453	0.17	2 691 444	11.55	7321	0.11	3 271 601	13.14	8825	0.13	3 740 846	14.83
snRNA	3817	0.06	9709	0.04	4754	0.08	17 003	0.07	3634	0.06	9779	0.04	4130	0.06	15 089	0.06
snoRNA	1862	0.03	4032	0.02	3490	0.06	10405	0.04	2202	0.03	6337	0.03	2709	0.04	7594	0.03
miRNA	46 961	0.71	3 974 298	17.30	42 070	0.69	5 638 271	24.19	44 071	0.68	4 795 634	19.27	46 018	0.68	4 031 156	15.98
Unann	6 475 993	97.90	17 067 997	74.28	5 976 291	97.50	14 080 950	60.42	6 385 673	98.04	16 163 653	64.94	6 642 704	97.83	16 530 545	65.54
Total	6 614 810		22 976 449		6 129 644		23 305 356		6 513 515		24 889 363		6 790 318		25 222 128	

S, sensitive; T, tolerant; C, control; D, drought; Mapped, mapped to tomato genome (ITAG Release 2.4); Unann, unannotated tags.

### Identification of miRNAs from deep sequencing

To identify miRNAs, the clean sequence tags were aligned to all plant miRNA mature sequences deposited in miRBase database (Kozomara and Griffiths‐Jones, 2014). The average of 44 046 (0.72%) and 44 780 (0.69%) unique, and 2 157 079 (8.94%) and 4 609 840 (19.19%) redundant reads were matched to the currently known miRNA sequences for root and upground libraries, respectively (Tables 1 and 2). Totally, 699 miRNAs were obtained from sequencing in eight libraries which belong to 578 families (Table S2). Among 578 families, sly‐miR171 was represented with seven members, as the largest one, followed by sly‐miR166 and sly‐miR319 with five members (Table S2). Eleven miRNA families (sly‐miR156, sly‐miR157, sly‐miR164, sly‐miR166, sly‐miR167, sly‐miR168, sly‐miR4414, sly‐miR6022, sly‐miR6027, sly‐miR7822 and sly‐miR9471) were represented with the top read abundance above 10 000 at all libraries (Table S2).

**Figure 2:**
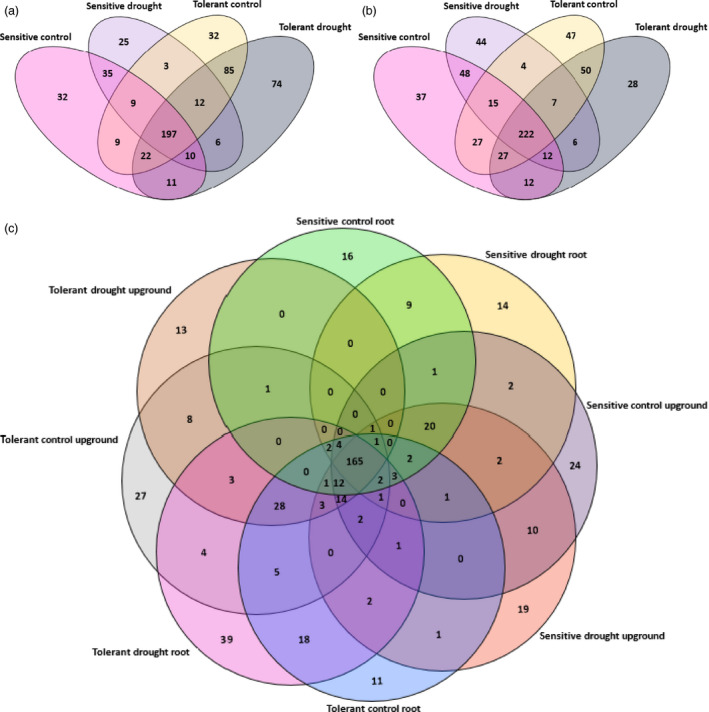
Distribution of tomato miRNAs (a) in root tissues, (b) in upground tissues, (c) in all samples.

We evaluated the miRNA distribution among libraries and determined that 197 miRNAs were common among control and drought‐treated samples of both sensitive and tolerant genotypes in root libraries (Figure 2a). When we compared the genotypes separately, 35 miRNAs were common between control and drought samples of sensitive genotype and 32 miRNAs such as sly‐miR166k, sly‐miR408‐3p and sly‐miR9552b‐3p were specific to control plants, whereas 25 miRNAs such as sly‐miR1101‐3p, sly‐miR2628 and sly‐miR3932b‐3p were expressed only in drought‐treated roots of sensitive genotype (Table S2). In tolerant genotype, 32 miRNAs such as sly‐miR165b‐5p, sly‐miR2867‐3p and sly‐miR7520 were expressed only in control conditions, while 74 miRNAs such as sly‐miR171f‐5p, sly‐mir838‐5p and sly‐miR8046‐3p were expressed specifically after drought exposure (Table S2). Additionally, 85 miRNAs were commonly expressed between control and drought treatments in tolerant genotype (Figure 2a). In upground samples, 222 miRNAs were common among four samples. Additionally, in control and drought‐treated samples, 37 miRNAs such as sly‐miR171a‐3p, sly‐miR6024‐3p and sly‐miR7997a were expressed specifically in control conditions, while 44 miRNAs such as sly‐miR845a, sly‐miR5797 and sly‐miR8762d were specific to drought‐treated upground tissues in sensitive genotype (Table S2, Figure 2b). In terms of tolerant genotype, 47 miRNAs such as sly‐miR166d‐5p, sly‐miR4392 and sly‐miR6288a showed specific expression in control sample, whereas 28 miRNAs such as sly‐miR904a, sly‐miR5171a and sly‐miR6485 were expressed specifically in response to dehydration stress (Table S2, Figure 2b). Furthermore, sensitive control and drought libraries shared 48 miRNAs, while tolerant control and drought libraries had 50 common miRNAs (Figure 2b). When we compared eight libraries consisting of 699 miRNAs, we determined 165 miRNAs to be expressed commonly in all samples, while 63 and 90 miRNAs were expressed only in sensitive (C‐R, 16; D‐R, 14; C‐U, 24; D‐U, 19) and tolerant genotypes (C‐R, 11; D‐R, 39; C‐U, 27; D‐U, 13), respectively (Figure 2c).

### Expression analyses of miRNAs

**Figure 3:**
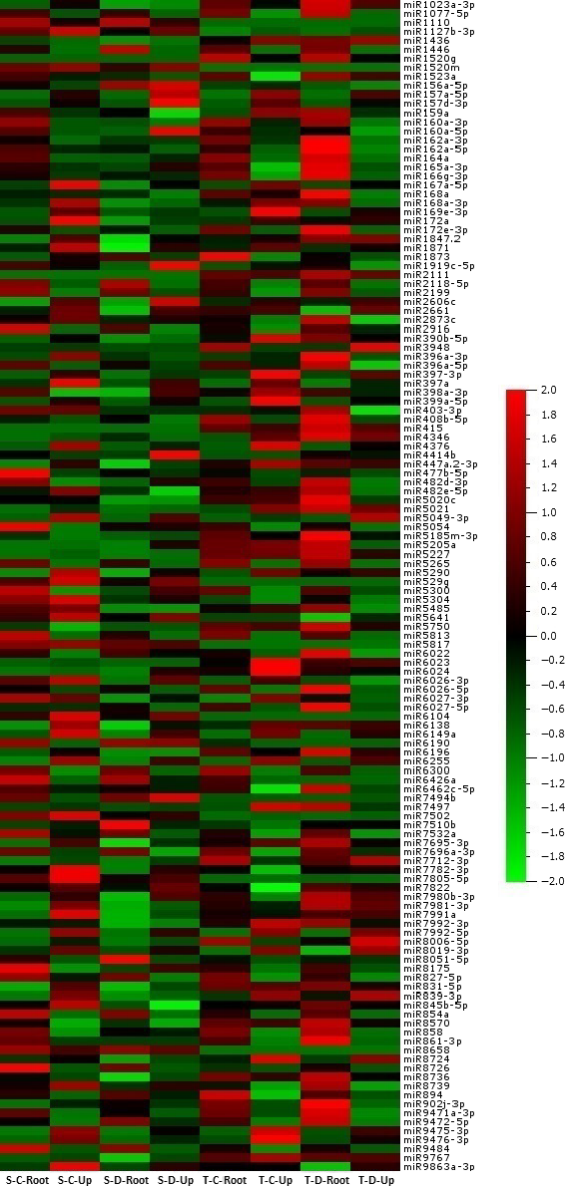
Differential expression of 130 most abundant conserved miRNAs in drought‐sensitive and drought‐tolerant tomato roots and upgrounds. The miRNA expressions were shown as *Z*‐score. S, sensitive; T, tolerant; C, control; D, drought; Up, upground.

**Figure 4:**
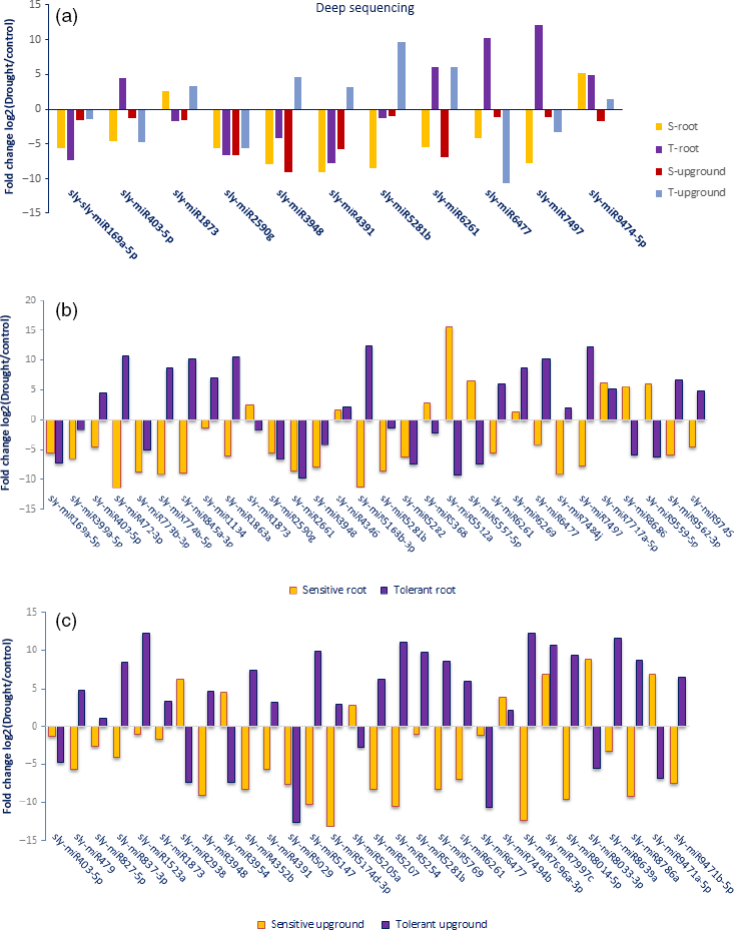
Comparisons of conserved miRNA expression changes after drought exposure according to the deep sequencing results. (a) Common miRNAs among root and upground tissues of sensitive and tolerant genotypes; (b) Common miRNAs only in root tissues of sensitive and tolerant genotypes; (c) Common miRNAs only in upground tissues of sensitive and tolerant genotypes.

**Figure 5:**
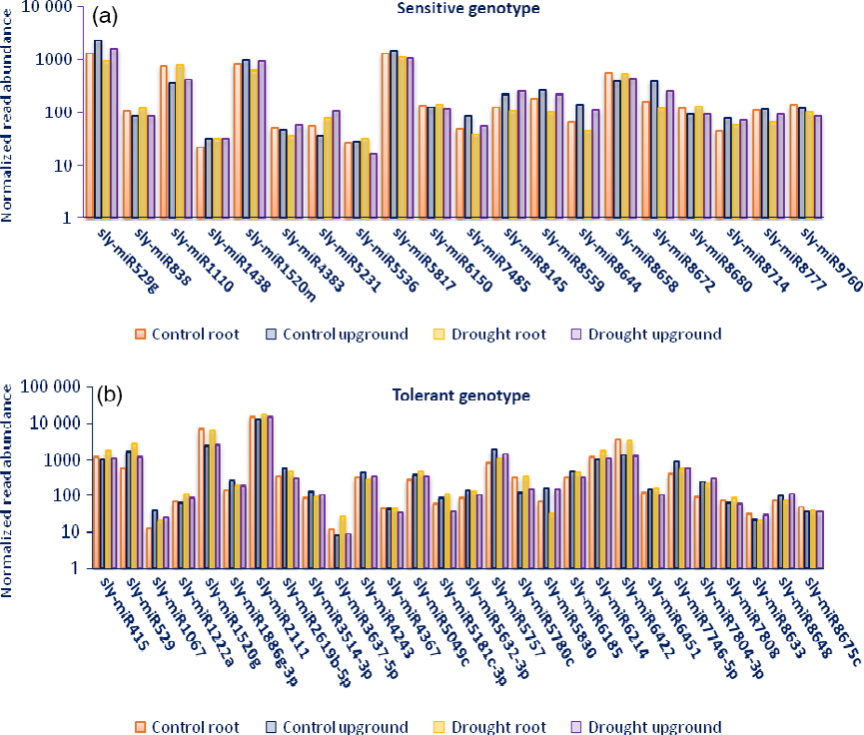
Normalized read abundance comparisons of conserved miRNAs belong to control and drought‐treated root and upground tissues specific to (a) sensitive genotype, (b) tolerant genotype.

A total of 688 of 699 (98.4%) miRNAs belonging to eight libraries were expressed significantly based on fold change (≥1 or ≤−1) and *P*/*q*‐value (<0.05) criteria (Table S2) and at least 130 miRNAs expressed approximately in all tomato libraries (Figure 3). Some miRNAs expressed differentially in root and upground tissues of sensitive and tolerant genotypes under control and drought conditions. Generally, the majority of miRNAs were down‐regulated in sensitive genotype (mostly in upgrounds), while up‐regulated in tolerant genotype (mostly in roots) by drought stress treatment (Table S2). A total of 11 miRNAs showed significant expression in all tissues of two genotypes in response to drought stress (Figure 4a, Table S2). For example, the expression of sly‐miR169a‐5p was decreased in all tissues, even the decrease was higher in root tissues. However, the expression of sly‐miR6261 was decreased in root and upground tissues in sensitive genotype, whereas increased in tolerant genotype. In root, specific expression changes were observed. Some miRNAs were down‐regulated in sensitive genotype, while up‐regulated in tolerant by drought exposure; these miRNAs include sly‐miR403‐3p and sly‐miR845a‐3p. Contrary to this, sly‐miR5512a and sly‐miR9559‐5p were up‐regulated in sensitive genotype, but down‐regulated in tolerant genotype. Additionally, the expression patterns were similar for certain miRNAs in both sensitive and tolerant genotypes. For instance, sly‐miR399a‐5p and sly‐miR5282 were down‐regulated with drought, while sly‐miR4346 and sly‐miR6269 were up‐regulated in root tissues of two genotypes (Figure 4b, Table S2). Similarly, same expression alteration patterns were observed in upground tissues. For example, the expressions of sly‐miR7494b and sly‐miR7997c were increased, whereas sly‐miR5029 were decreased in two genotypes. In addition, some adverse alterations were observed in two genotypes. Sly‐miR479 and sly‐miR837‐3p were down‐regulated, but up‐regulated in sensitive and tolerant genotypes, respectively; sly‐miR3954 and sly‐miR9471a‐5p were up‐regulated, but down‐regulated in sensitive and tolerant genotypes, respectively (Figure 4c, Table S2). Some miRNAs showed tissue‐ and genotype‐specific expression patterns. For example, several miRNAs were expressed only in root tissues in sensitive genotype and repressed/downregulated by drought exposure such as sly‐miR166k, sly‐miR408‐3p and sly‐miR9552b‐3p (Table S2). Similarly, some miRNAs showed expression only in upground tissues of sensitive genotype and the expression level was decreased or repressed after drought stress such as sly‐miR171a‐3p, sly‐miR1426, sly‐miR5239, sly‐miR6024‐3p and sly‐miR7997a (Table S2). In tolerant genotype, the expression of some miRNAs (sly‐miR2867‐3p, sly‐miR3514‐5p, sly‐miR5251, sly‐miR5763, sly‐miR7520, sly‐miR7730‐5p, sly‐mi8751b and sly‐miR9493) was also specific to root tissues and suppressed/decreased by dehydration (Table S2). Likewise, some miRNAs were expressed specifically in drought‐tolerant upground tissues and down‐regulated or repressed with drought treatment such as sly‐miR166d‐5p, sly‐miR408a‐3p, sly‐miR1507c‐5p, sly‐miR1857‐5p, sly‐miR4392, sly‐miR6288a and sly‐miR9722 (Table S2). Certain miRNAs were induced/upregulated by drought treatment in all tissues and genotypes. Among them, sly‐miR319a, sly‐miR1101a‐3p, sly‐miR2628 and sly‐miR3932b‐5p were specific to sensitive root tissues; sly‐miR845a, sly‐miR1511‐3p, sly‐miR5259, sly‐miR5797 and sly‐miR6440b to sensitive upground tissues; sly‐miR171f‐5p, sly‐miR838‐5p, sly‐miR946a‐5p, sly‐miR1846a‐5p, sly‐miR3637‐3p, sly‐miR5035‐3p, sly‐miR5760 and sly‐miR6172 to tolerant root; and sly‐miR904a, sly‐miR5171a, sly‐miR6173, sly‐miR6225‐3p and sly‐miR6485 to tolerant upground tissues (Table S2). When we evaluated the drought‐sensitive and tolerant genotypes, we determined that a total of 20 miRNAs were expressed in all four libraries belonging to sensitive genotype such as sly‐miR529 g and sly‐miR5817 (Figure 5a, Table S2). Similarly, 28 miRNAs showed expression only in all tolerant‐related libraries such as sly‐miR415, sly‐miR1520 g and sly‐miR2111 (Figure 5b, Table S2).

### Target prediction and degradome analyses

psRNATarget server (http://plantgrn.noble.org/psRNATarget/) was used to predict miRNA target transcripts; a total of 41 300 miRNA target pairs were obtained which contain 688 significantly expressed miRNAs (Table S3). These targets were mainly transcription factors, stress‐ and dehydration‐/drought‐related proteins, resistance‐associated proteins and significant cellular enzymes like kinases, transferases and phosphatases (Table S3). Among these, 360 miRNAs (such as sly‐miR319‐3p, sly‐miR2089‐3p and sly‐miR5671a) targeted plant development and stress response‐related transcription factors, such as MYB, WRKY, GRAS, TCP, NAC‐domain, ARF (auxin response factor), SBP (squamosa promoter‐binding‐protein‐like), LEA (late embryogenesis abundant) protein and ERF (ethylene responsive factor) families (Table S3). Additionally, stress‐related proteins known as stress‐responsive protein, stress‐enhanced protein, universal stress protein and stress‐induced protein were potentially targeted by 49 miRNAs, such as sly‐miR164a, sly‐miR1074, sly‐miR1873, sly‐miR2628 and sly‐miR5029. Also, especially some miRNA target genes were associated with dehydration/drought stress directly. These transcripts included dehydration‐responsive family protein, DRP (dehydration‐responsive protein), ERD (early responsive to dehydration‐like) protein, DREB (dehydration‐responsive element binding) and Di19 protein (dehydration‐/drought‐induced 19 protein) and potentially regulated by 38 tomato miRNAs; these miRNAs included sly‐miR160a‐3p, sly‐miR170‐3p, sly‐miR1074, sly‐miR3948, sly‐miR5081, sly‐miR5758, sly‐miR8001b‐5p and sly‐miR9748.

**Figure 6:**
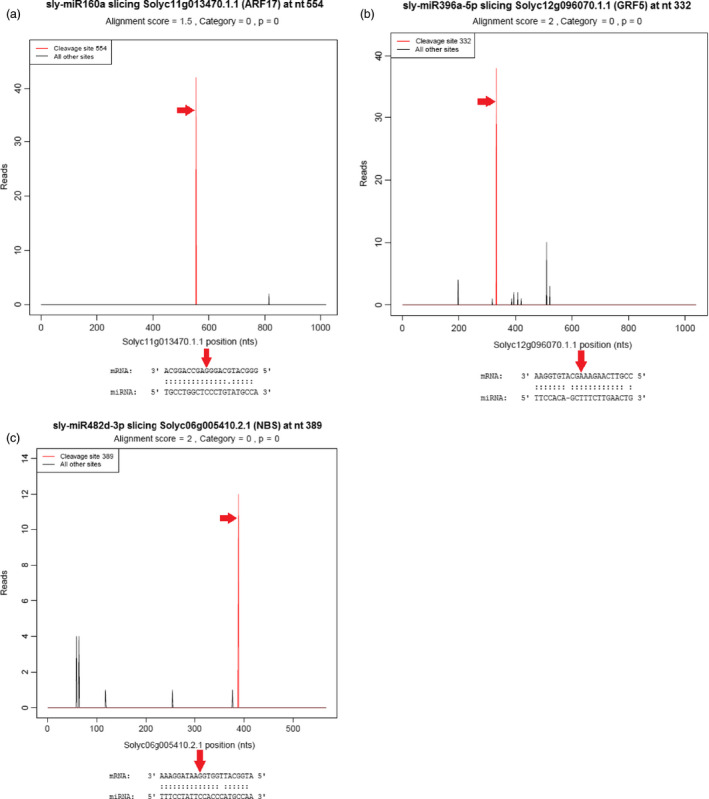
Target (T) plots and miRNA–mRNA alignments of tomato. (a) sly‐miR160a cleaves ARF17 gene; (b) sly‐miR396a‐5p cleaves GRF5 gene; (c) sly‐miR482d‐3p cleaves NBS protein. The red arrows represent the cleavage nucleotide positions on the target genes.

We employed degradome sequencing to identify miRNA targets. After sequencing, a total of 10 819 148 raw and 10 799 028 clean tags (99.81%) were obtained. Then, the clean tags were mapped to the reference genome database of tomato ITAG 2.4 Release (ftp://ftp.solgenomics.net/genomes/Solanum_lycopersicum/annotation/ITAG2.4_release/ITAG2.4 genomic.fasta) by SOAP2.20 (Li *et al*., 2009) and 7 422 369 matched tags (68.73%) were determined. With the classification of these tags, cDNA_sense tags were selected and the identified 1 249 158 (46.86%) unique 5′ cDNA tags were used for the prediction of cleavage sites of tomato miRNAs. After prediction using CleaveLand v3.0.1 pipeline (Addo‐Quaye *et al*., 2009), a total of 59 cleavage sites were determined associated with 62 miRNAs and 44 target genes and 115 specific miRNA–mRNA pairs were predicted at cleavage sites with *P*‐value <0.05 (Table S4). For the identification of cleavage sites, degradome peaks are classified into five categories according to the peak height at mRNA position and the targets with category 0 or 1 were evaluated as the most significant (Karlova *et al*., 2013). In tomato degradome results, 15 target genes were related to stimulus response such as ARF and disease resistance proteins were identified in category 0 cleaved by sly‐miR160, sly‐miR168, sly‐miR172, sly‐miR396, sly‐miR482, sly‐miR6023 and sly‐miR6024 families (Figure 6, Table S4). In category 1, only two genes cleaved by miR156 and miR162 families were obtained (Table S4). In another significant class of peaks, category 2, mostly stimulus‐ and cellular component organization‐associated 47 mRNAs like PSII (photosystem II) protein, NAD(H) kinase, phosphorus transporter, ATP‐sulfurylase and SCL (scarecrow‐like) protein were determined. These targets were cleaved by sly‐miR156, sly‐miR164, sly‐miR166, sly‐miR169, sly‐miR171, sly‐miR395 and sly‐mir9477 (Table S4). The rest of 51 target genes were belonged to less significant categories 3 and 4. One of the stimulus response‐associated target PSII degraded by 16 miRNA families such as sly‐miR167, sly‐miR319, sly‐miR390, sly‐miR482, sly‐mir1919, sly‐miR5302 and sly‐miR9479. Other two stress‐related genes, SBP and ARF, were cleaved by sly‐miR156 and sly‐miR160 families, respectively. Sly‐miR171, sly‐miR403 and sly‐miR6027 families targeted histone‐arginine methyltransferase involved in cellular and developmental process, and sly‐miR395 and sly‐miR9477 degraded acyltransferase gene, the cellular and metabolic process‐associated target (Table S4).

### GO enrichment and KEGG pathway analyses

**Figure 7:**
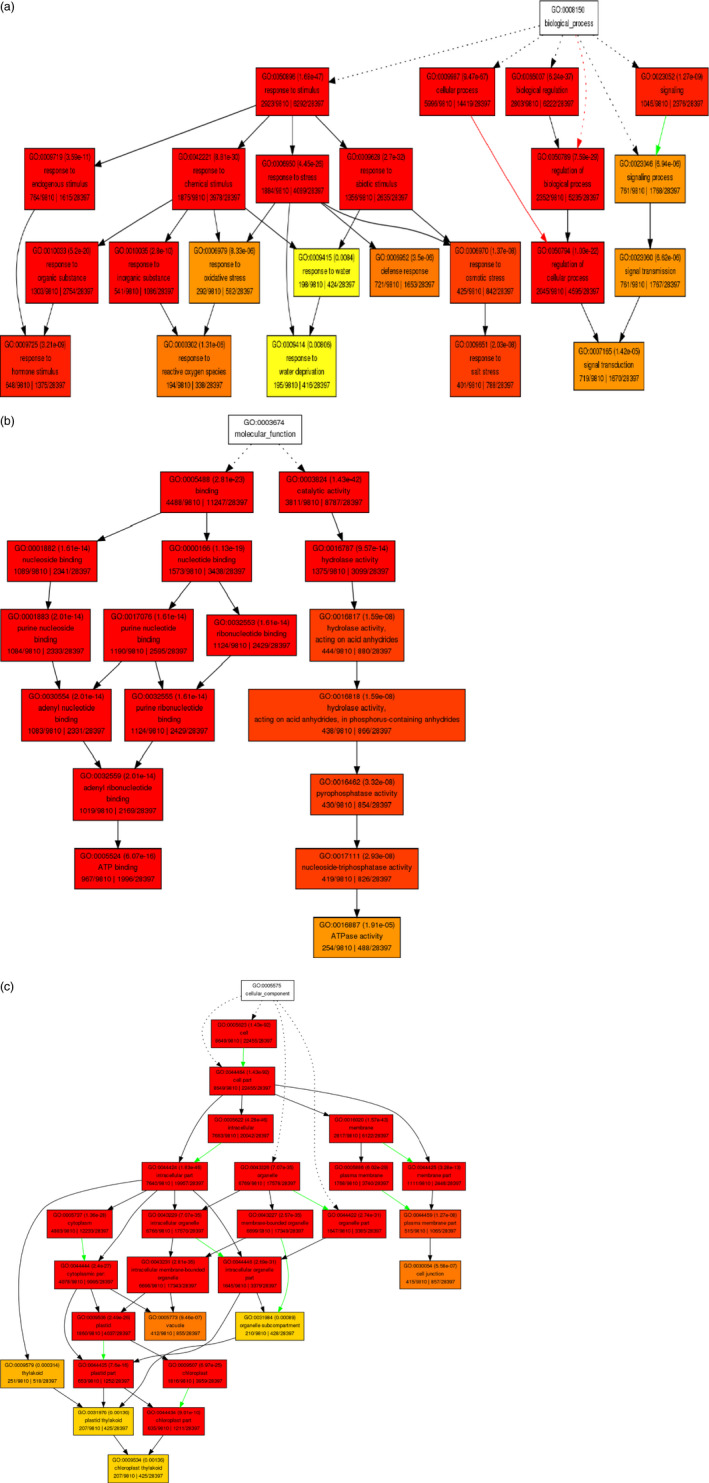
Diagrams of enriched GO terms of drought‐responsive miRNA‐associated tomato targets constructed by AGRIGO. (a) biological process, (b) molecular function and (c) cellular component. Red to yellow colours represent decreasing significance levels (Red is most, yellow is least significant). Red and green arrows mean positive and negative regulation of terms.

**Figure 8:**
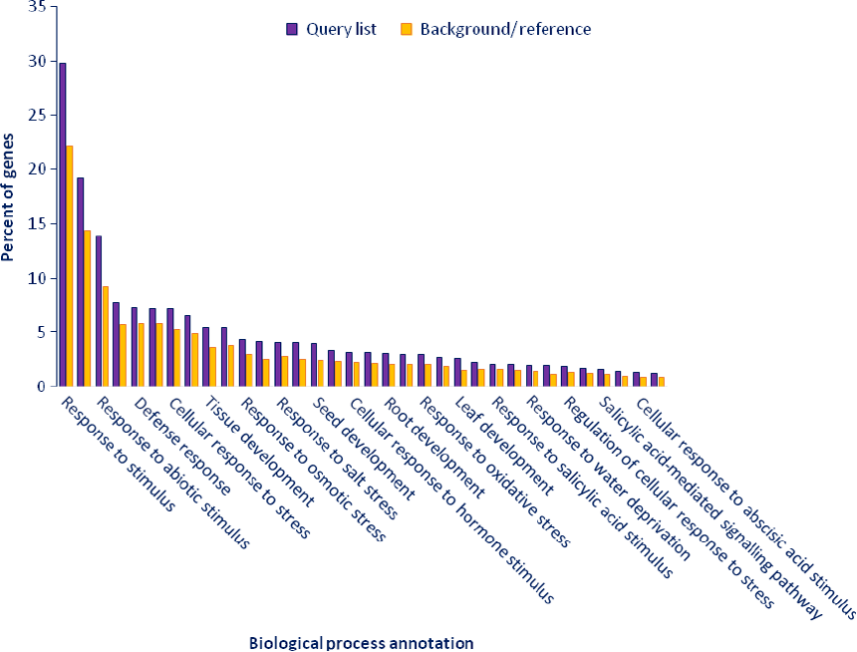
Functional annotation of GO biological process related with drought stress in tomato by AGRIGO.

Gene ontology (GO)‐based enrichment analysis was performed to further investigate the potential role of miRNAs in tomato response to drought stress. A total of 9810 potential miRNA targets were classified into 665 biological processes, 45 molecular functions and 72 cellular components (Table S5). Some of the significant biological processes with the highest target numbers were cellular process (GO:0009987), cellular metabolic process (GO:0044237), response to stimulus (GO:0050896), response to abiotic stimulus (GO:0009628), response to stress (GO:0006950) and response to water deprivation (GO:0009414) (Table S5). Binding (GO:0005488), catalytic activity (GO:0003824), nucleotide binding (0000166) and hydrolase activity (0016787) were among the most abundant classes in molecular function category (Table S5). The common cellular component terms were cell part (GO:0044464), intracellular part (GO:0044424), organelle part (GO:0043226) and cytoplasmic part (GO:0044444) (Table S5). We employed agriGO web‐based tool to visualize the enriched biological process, molecular function and cellular component categories and draw hierarchical graphs of significantly enriched GO terms (Du *et al*., 2010). In biological process, stress‐associated terms such as response to hormone stimulus (GO:0009725) like abscisic acid (GO:0009737), jasmonic acid (GO:0009753), auxin (GO:0009733), ethylene (GO:0009723) and salicylic acid (GO:0009751), response to reactive oxygen species (GO:0000302), response to water deprivation (GO:0009414), response to salt stress (GO:0009651) and signal transduction (GO:0007165) were related to each other (Figures 7a and 8) and development‐related ontologies like fruit development (GO:0010154), shoot development (GO:0048367), seed development (GO:004831), root development (GO:0048364) and leaf development (GO:0048366) were determined (Figure 8). Pyrophosphatase activity (GO:0016452), nucleoside‐triphosphatase activity (GO:0017111), ATPase activity (GO: 0016887), purine ribonucleotide binding (GO: 003255) and ATP binding (GO:0005524) were some of the significantly enriched molecular function terms (Figure 7b, Table S5). In cellular component category, organelle subcompartment (GO:0031984), cell junction (GO:0030054), vacuole (GO:0031984) and chloroplast thylakoid (GO:0009534) were categorized significantly (Figure 7c, Table S5).

**Figure 9:**
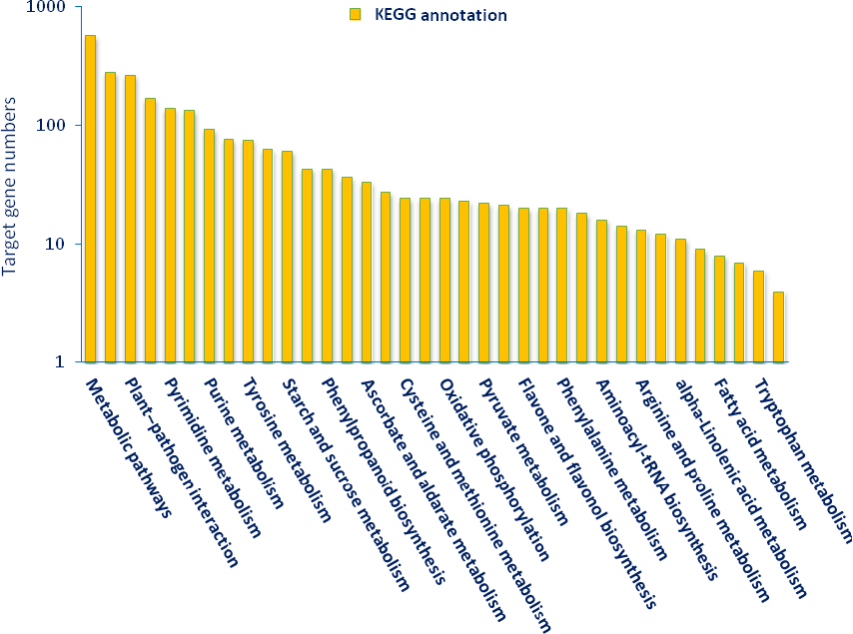
Functional annotation of KEGG pathways in tomato by KEGG database.

**Figure 10:**
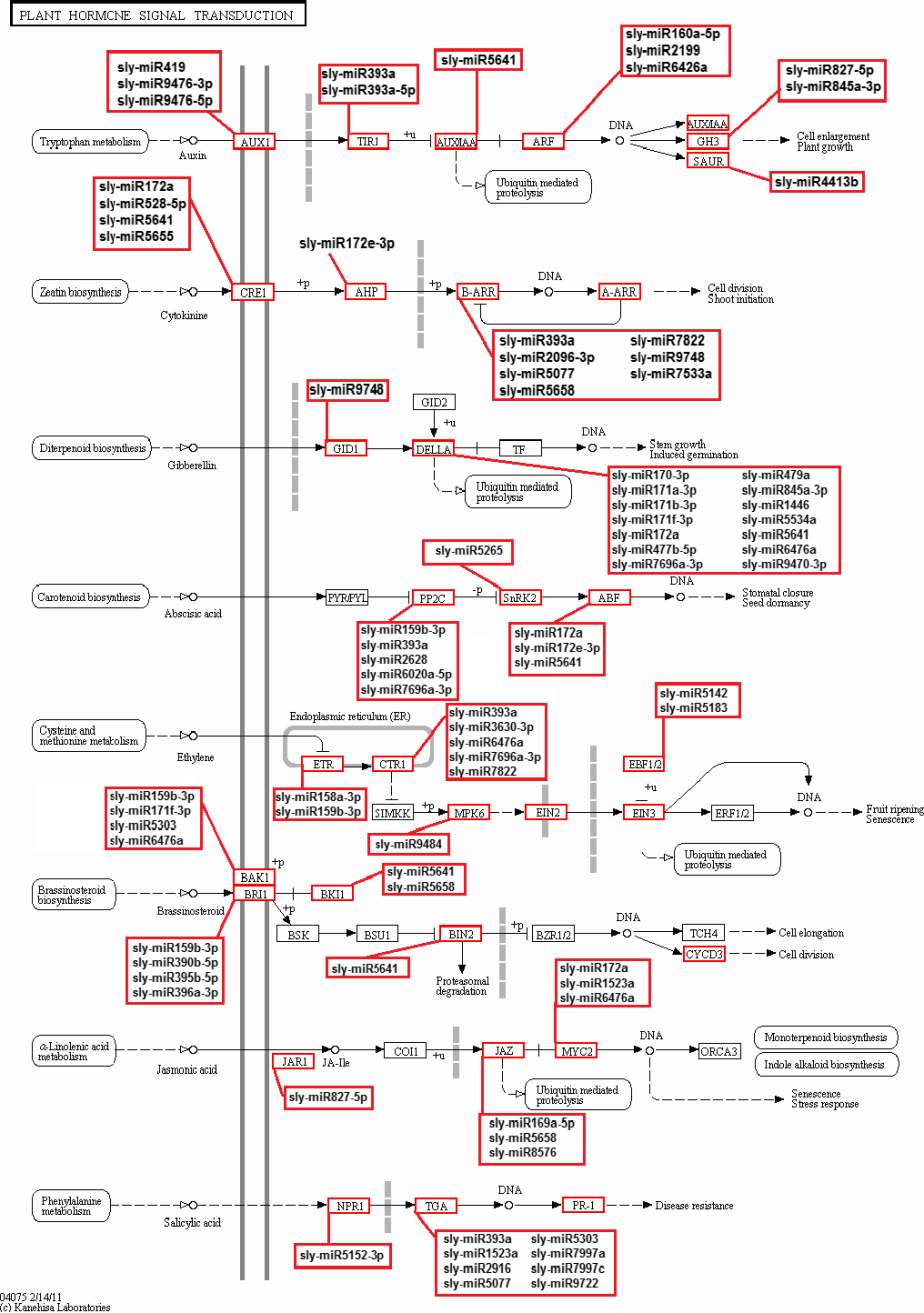
Plant hormone signal transduction pathway and related miRNAs.

KEGG (The Kyoto Encyclopedia of Gene and Genome) annotation was carried out for pathway analysis, and 289 KEGG pathways were obtained. Thirty‐six pathways including 2414 genes were detected as closely associated with drought stress including plant–pathogen interaction, biosynthesis of plant hormones, plant hormone signal transduction, oxidative phosphorylation, calcium signalling pathway, carotenoid metabolism, alpha‐Linolenic acid metabolism, brassinosteroid biosynthesis and photosynthesis (Figure 9). Plant hormone signal transduction pathway was represented by 134 genes and contained auxin‐, cytokinine‐, gibberellin‐, abscisic acid‐, ethylene‐, brassinosteroid‐, jasmonic acid‐ and salicylic acid‐associated signalling genes inducible via abiotic and biotic stresses (Figure 10). For example, carotenoid biosynthesis was involved in the pathway, contributes to abscisic acid synthesis, contained enriched phosphatase 2C (PP2C), plant‐specific serine/threonine kinase (SnRK2) and abscisic acid‐responsive element binding factor (ABF). These genes were regulated by sly‐miR172a, sly‐miR172e‐3p, sly‐miR393a, sly‐miR2628, sly‐miR5265, sly‐miR5641, sly‐miR6020a‐5p and sly‐miR7696a‐3p (Figure 10). Similarly, jasmonic acid–amido synthetase JAR1, jasmonate ZIM domain (JAZ) and MYC2 genes were enriched to alpha‐Linolenic acid metabolism associated with jasmonic acid synthesis, and these genes were targeted by sly‐miR162a‐3p, sly‐miR169a‐5p, sly‐miR172a, sly‐miR827‐5p, sly‐miR5083, sly‐miR5298a, sly‐miR5658, sly‐miR6476a and sly‐miR8576 (Figure 10).

**Figure 11:**
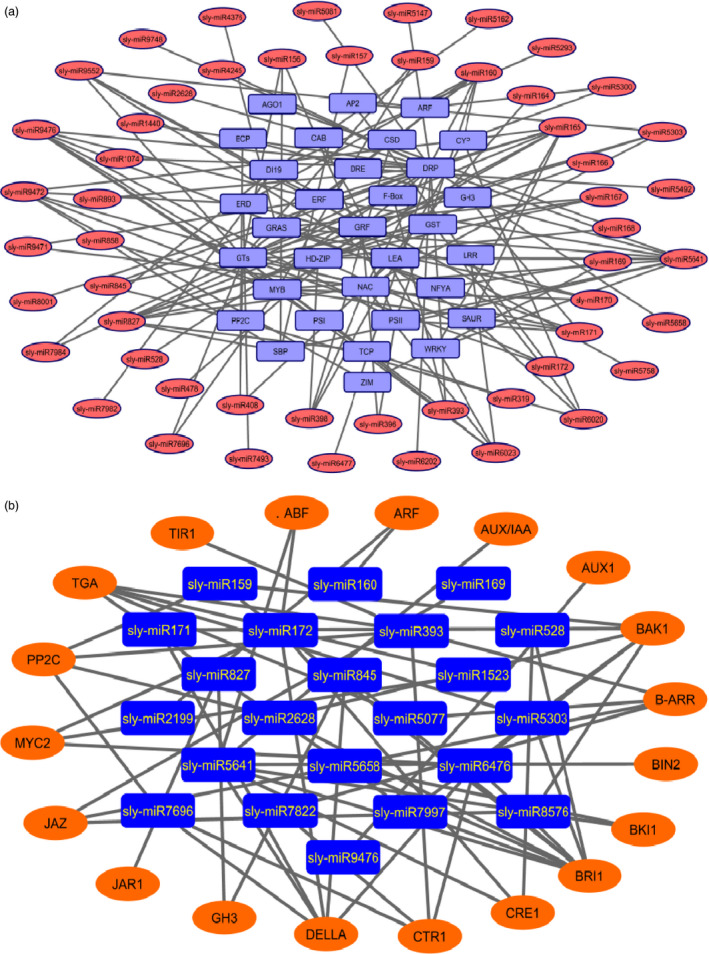
Relationships between miRNAs and their targets associated with (a) drought response, (b) plant hormone signal transduction pathway.

Cytoscape platform was further employed to build the network between the drought‐responsive miRNAs and their targets. Totally, 53 miRNAs (such as sly‐miR164, sly‐miR166 and sly‐miR408) targeted 23 genes associated with drought response and tissue development, which included NAC‐domain, HD‐ZIP and BCP (blue copper protein). Sly‐miR5641 targeted 10 drought‐ and development‐related genes such as DRP, LEA, ERF and SBP (Figure 11a). Different gene families were targeted by different numbers of miRNAs. For example, DRP was targeted by 20 miRNAs, followed by GTs (glycosyltransferases) with 16 miRNAs; MYB with 11 miRNAs; NAC with 8 miRNAs; HD‐ZIP with 7 miRNAs; and PSII and ERF with 6 miRNAs (Figure 11a). In plant hormone signal transduction pathway, 23 miRNAs targeted 19 genes that play roles in cell division, plant growth, stomatal closure and stress response. For example, sly‐miR5641 potentially targeted seven genes involved in this pathway. Additionally, sly‐miR393/miR6476 and sly‐miR172/miR5658 potentially targeted 5 and 4 related genes, respectively (Figure 11b).

### qRT‐PCR validation of miRNA expressions

**Figure 12:**
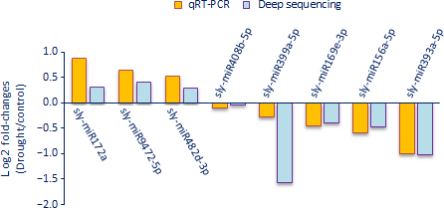
qRT‐PCR validation of randomly selected drought‐responsive eight miRNAs in tolerant root tissues.

We performed qRT‐PCR to validate the deep sequencing results with randomly selected eight miRNAs (sly‐miR156a‐5p, sly‐miR169e‐3p, sly‐miR172a, sly‐miR393a‐5p, sly‐miR399a‐5p, sly‐miR408b‐5p, sly‐miR482d‐3p and sly‐miR9472‐5p). For this purpose, we used root tissues of tolerant genotype under control and drought conditions and we figured out the expression levels between drought‐treated versus control samples using log2 fold change ($2^{-\Delta \Delta C_{t}}$) values with three technical and three biological replicates. The expression results of root tissues in tolerant genotype exposed to drought were similar to the deep sequencing data (Figure 12). Three miRNAs (sly‐miR172a, sly‐miR482d‐3p and sly‐miR9472‐5p) were up‐regulated in qRT‐PCR analysis showing a positive correlation with deep sequencing results. Similarly, the other miRNAs were down‐regulated in both qRT‐PCR and high‐throughput sequencing results (Figure 12).

## Discussion

MicroRNAs, the post‐transcriptional gene regulators, are associated with drought stress evidenced by that miRNAs target a wide range of drought‐related genes, such as ARF, MYB, TCP and GRAS family transcription factors, dehydrins, glutathione S‐transferase (GST) and abscisic acid‐related genes (Ferdous *et al*., 2015). Using microarray and deep sequencing approaches, several drought‐responsive miRNAs were identified in wheat (Kantar *et al*., 2011), sorghum (Pasini *et al*., 2014), sugarcane (Thiebaut *et al*., 2014), tobacco (Yin *et al*., 2014), potato (Zhang *et al*., 2014) and barley (Hackenberg *et al*., 2015). However, there is no study on systematical identification and expression analysis of miRNAs in tomato response to drought stress using microarray or deep sequencing approaches. In this study, we employed deep sequencing technology to systematically study the effect of drought exposure on miRNA expression in two different tomato genotypes with different drought sensitivity in a tissue‐specific manner. Based on our study, a total of 775 miRNAs were differentially affected by drought; among them, 438 miRNAs were down‐regulated in sensitive genotype (Table S2). We also performed degradome sequencing to identify miRNA targets. Our results were different from that in tomato fruit development (Karlova *et al*., 2013). In our study, we found 44 target genes, but only the targets of 4 miRNAs (sly‐miR156, sly‐miR160, sly‐miR166 and sly‐miR482) were matched with their results (Table S4). We identified not only the common targets as reported previously but also new targets in tomato. For example, miR156 targets SBP transcription factors, but sly‐miR156d‐3p targeted NAD(H)‐like kinase protein, while sly‐miR156e‐5p targeted four genes including SBP6 and PSII protein. Except the previous report that miR160 was found to target ARF10, ARF16 and ARF17 (Karlova *et al*., 2013), we also found that miR160 targeted ARF18‐like and PSII protein (Table S4). Several other drought‐related miRNAs targeted several new genes.

Despite the similarity of each member in a same miRNA family (Zhang *et al*., 2006), they may response differently to drought stress with a tissue‐ and genotype‐dependent manner (Table S2). For example, miR165/166 families were known to target HD‐ZIP III transcription factor which is crucial for leaf polarity, lateral root development and vascular patterning (Elhiti and Stasolla, 2009; Williams *et al*., 2005; Zhong and Ye, 2007). The expressions of miR165/166 family were generally decreased by drought treatment in other plant species such as rice (Zhou *et al*., 2010), cotton (Xie *et al*., 2015), wheat (Kantar *et al*., 2011) and peach (Eldem *et al*., 2012). However, the expression of miR166 was increased in *Medicago truncatula* by drought stress and the expression level was higher in roots in comparison with upper parts (Trindade *et al*., 2010). Moreover, overexpressions of miR165/166 caused formations of vascular bundles and decreased lateral roots, respectively (Boualem *et al*., 2008; Zhong and Ye, 2007). These results suggest that miR165/166 families regulate root development and drought response. In our study, drought majorly induced the expression of miR165, but inhibited the expression of miR166 in roots. Additionally, sly‐miR165a‐5p/miR166k and sly‐miR165b‐5p were root specific in sensitive and tolerant genotypes, respectively, whereas sly‐miR166d‐5p was specific to upground tissues of tolerant genotype. Drought did not affect the sensitive genotype significantly in miR165 family. However, in root tissues of tolerant genotype, the expression of miR165b‐5p was decreased by −8.25‐fold after drought treatment, whereas miR165a‐3p was up‐regulated (3.10‐fold) in upground tissues. Sly‐miR166a and sly‐miR166 g‐3p were up‐regulated by 19.50‐ and 2.34‐fold, respectively, in upground tissues of tolerant genotype with dehydration, while miR166d‐5p expression was decreased sharply (−10.98‐fold). In sensitive root samples, sly‐miR166 g‐5p was up‐regulated (11.64‐fold) by drought stress, whereas miR166k expression was decreased at the same time by −18.71‐fold (Table S2). These results suggest that miR165 and miR166 regulated concurrently the drought‐responsive gene expression as positively or negatively.

One of the main targets of miR398 is copper/zinc superoxide dismutase (Cu/Zn‐SOD, CSD), a scavenger enzyme of ROS (reactive oxygen species) detoxifying superoxide radicals (Sunkar *et al*., 2006). miR398 was down‐regulated by drought in tomato (Luan *et al*., 2014), *M. truncatula* (Wang *et al*., 2011) and cotton (Xie *et al*., 2015), whereas up‐regulated in another *M. truncatula* species (Trindade *et al*., 2010) and wheat (Kantar *et al*., 2011). Down‐regulation of this miRNA results in increase in CSD expression and tolerance to oxidative stress (Ding *et al*., 2013). Consistently, the overexpression of miR398 led to down‐regulation of CSD1 and 2 enzymes and caused sensitivity to drought stress in rice (Lu *et al*., 2010). Our target identification results were consistent with the literature and sly‐miR398a‐3p targeted CSD enzyme also (Table S3). However, sly‐miR398a‐3p expression did not change significantly by drought (Figure 3, Table S2). Sly‐miR398a‐5p was down‐regulated in sensitive upground and tolerant root tissues by 2.65‐ and 1.17‐fold, respectively, with drought treatment (Table S2). This miRNA targeted the development and drought‐related NAC‐domain, HD‐ZIP III and auxin response proteins according to the target prediction results (Table S3). Sly‐miR398a‐5p expressed higher in roots of sensitive genotype in control (1.29‐fold) and drought (4.09‐fold) conditions, but generally miR398 family expression was decreased in upground tissues excluding sly‐miR398a‐3p in sensitive genotype. This up‐regulation (2.03‐fold) may be responsible for the sensitivity of *L. esculentum* decreasing CSD activity. The other copper proteins plantacyanin (basic blue) and laccase were the predicted targets of miR408 family (Abdel‐Ghany and Pilon, 2008), and the expression of this family usually decreased by dehydration in plant species such as rice (Zhou *et al*., 2010), *M. truncatula* (Trindade *et al*., 2010), *Populus* (Ren *et al*., 2012), peach (Eldem *et al*., 2012) and cotton (Xie *et al*., 2015). miR408 is necessary for the adjustment of copper levels in cells as the copper deficiency causes production of ROS and oxidative stress (Abdel‐Ghany and Pilon, 2008). The overexpression of miR408 in chickpea led to the inhibition of plantacyanin expression and accumulation of copper and also induction of DREB expression by drought (Hajyzadeh *et al*., 2015) and overexpression of DREB transcription factors increased drought tolerance in rice (Chen *et al*., 2008) and *Arabidopsis* (Xu *et al*., 2009). According to our results, miR408a‐3p targeted laccase protein as well as other drought‐related genes GTs and LEA proteins (Table S3). The expression of miR408 was decreased after drought treatment. Especially, miR408‐3p was down‐regulated by −10.09‐fold in root tissues of sensitive genotype and this miRNA was suggested as root specific to sensitive genotype as it was expressed only in root tissues of *L. esculentum*. Besides, miR408a‐3p expression was decreased by −1.02‐fold in upground tissues of tolerant genotypes and was suggested as upground specific in *L. esculentum* var. *cerasiforme*. High‐level of decrease in miR408‐3p expression in *L. esculentum* may be the reason of drought sensitivity by comparison with miR408a‐3p expression change in tolerant genotype.

The expression of miR9552 showed drought‐, tissue‐ and genotype‐specific pattern. Sly‐miR9552a‐3p was only expressed in the roots of sensitive genotype and induced (12.38‐fold) by drought treatment, whereas repressed (−14.16‐fold and −13.64‐fold) in upground tissues of sensitive and tolerant genotypes (Table S2). miR9552 targeted SAUR protein whose expression is regulated by auxin (Abel and Theologis, 1996). Overexpression of SAUR39 gene caused the formation of shorter plants with less leaves in rice indicating negative correlation with auxin biosynthesis (Kant *et al*., 2009). In contrast, overexpression of SAUR19‐24 genes led to large leaves and hypocotyls implying cell enlargement and plant growth function of SAUR proteins induced by auxin (Spartz *et al*., 2012). Biotic and abiotic stress induced the differential expression of SAUR genes. For instance, 11 randomly selected SAUR genes were expressed in different tomato tissues and mostly down‐regulated by drought treatment (Wu *et al*., 2012). However, in our results, SAUR expression was found to be down‐regulated in root tissues, while up‐regulated in upground tissues contrary to miRNA expression profiles indicating tissue‐specific function of drought signalling in tomato. Sly‐miR9552b‐3p was expressed only in root tissues of sensitive genotype under control conditions and suppressed in response to stress treatment, so this miRNA might be suggested as root specific in *L. esculentum*. One of the predicted targets of this miRNA was UDP‐glucosyltransferase (UGT) (Table S3). Glycosyltransferase enzymes of plants (GTs, EC 2.4) function in secondary metabolism and hormone modification catalysing sugar addition to acceptor molecules such as auxin, ABA and flavonoids (Bowles *et al*., 2005; Tognetti *et al*., 2010) and play a role in biotic and abiotic stress tolerance (Vogt and Jones, 2000). One of the common glycosyltransferase UGT71C5 was investigated for the elucidation of ABA impact on drought adaptation in *Arabidopsis* plants, and when the gene was down‐regulated, drought tolerance increased but decreased after overexpression of UGT71C5 (Liu *et al*., 2015). These results suggest that the suppression of sly‐miR9552b‐3p expression with drought may increase UGT level in roots and decrease drought tolerance in sensitive genotype.

### Plant hormone signal transduction pathway

Plant hormones play a key role in signalling networks involving in plant development and stress response (Golldack *et al*., 2014). Different miRNAs regulate the expression of plant hormone‐associated genes in response to different environmental stresses. For example, a stress‐responsive gene ARF which is related with auxin signalling was targeted by sly‐miR160, sly‐miR2199 and sly‐miR6426 in response to drought stress in our study. However, ARF was targeted by miR167 after selenium treatment in *Astragalus* (Cakir *et al*., 2015). One of the important phytohormones, ABA, functions centrally in drought and salinity tolerance regulating main transcriptional processes (Cutler *et al*., 2010). The carotenoid biosynthesis of plant signal transduction pathway is regulated by ABA signals and these signals finally stimulate ABA‐responsive genes regulating the activation/inactivation of type 2C protein phosphatases (PP2Cs), SNF1‐related protein kinases (SnRK2s) and ABA‐responsive promoter elements binding factors (ABFs) (Golldack *et al*., 2014; Ma *et al*., 2009; Vlad *et al*., 2009). Our results found that PP2C was targeted by 5 miRNAs such as sly‐miR393a and sly‐miR7696a‐3p (Figures 10 and 11b). miR393 was up‐regulated in rice (Zhou *et al*., 2010), *Arabidopsis* (Liu *et al*., 2008) and wheat (Kantar *et al*., 2011), while down‐regulated in cotton (Xie *et al*., 2015) and peach (Eldem *et al*., 2012). Overexpression of miR393 led to decrease in drought tolerance affecting growth in rice (Xia *et al*., 2012). However, in this study sly‐miR393 was down‐regulated in root tissues of sensitive genotype, whereas up‐regulated in upground tissues of drought‐responsive tomato (Table S2). miR7696 also targeted ABA signalling pathway, whose expression was significantly altered in upground tissues of sensitive and tolerant genotypes and down‐regulated sharply by −12.33‐fold in sensitive genotype, whereas up‐regulated by 12.25‐fold in tolerant genotype after drought exposure. However, this miRNA was expressed higher in root tissues under control conditions (Figure 3, Table S2). These results indicate differential regulation of PP2C by several miRNAs in root and upground tissues of drought‐sensitive and tolerant genotypes. Similarly, ABF was targeted by sly‐miR172a/miR172e‐3p and sly‐miR5641 (Figures 10 and 11b). When the tomato plants were exposed to drought stress, miR172 family expressed significantly only in upground tissues of sensitive genotype and sly‐miR172a and sly‐miR172e‐3p were down‐regulated in response to drought by −2.01‐ and −1.07‐fold, respectively (Figure 3, Table S2). Sly‐miR172 expression was decreased after drought treatment in rice (Zhou *et al*., 2010), barley (Hackenberg *et al*., 2015) and cotton (Xie *et al*., 2015), whereas up‐regulated in *Arabidopsis* (Jones‐Rhoades and Bartel, 2004), wheat (Kantar *et al*., 2011) and *Populus* (Ren *et al*., 2012). Sly‐miR5641 was also down‐regulated by −4.31‐fold in root tissues of tolerant genotype (Figure 3, Table S2). The results show that miR172 is different in response to drought among plant species and ABF gene is regulated by different miRNAs in different tissues and genotypes under drought stress.

ABA usually interacts with gibberellic acid (GA) and jasmonate (JA) during plant development and response to drought stress (Golldack *et al*., 2014). GA signalling is controlled by GID1 (GIBBERELLIN INSENSITIVE DWARF1) receptors and DELLA proteins, the subgroup of GA‐responsive GRAS family transcription factors (Griffiths *et al*., 2006; Tyler *et al*., 2004). DELLA protein was targeted by 12 tomato miRNAs containing sly‐miR172, sly‐miR845, sly‐miR5641 and sly‐miR7696 (Figures 10 and 11b). miR845 was expressed differentially in two tissues and genotypes. Sly‐miR845a‐3p was down‐regulated (−8.86‐fold) in sensitive root tissues, whereas up‐regulated (10.09‐fold) in tolerant roots (Figures 3 and 4b, Table S2). In upground tissues, sly‐miR845a‐3p and miR845b‐5p were down‐regulated (~ −11.00‐fold), while miR845a was up‐regulated by 8.84‐fold (Figures 3 and 4c, Table S2). The results not only indicate the different regulatory roles of unique miRNA members in different tissues and genotypes, but also show the function of the miRNAs in more than one signalling way. In JA signalling, there are three key receptor proteins known as Jasmonate Resistant 1 (JAR1), Jasmonate ZIM Domain (JAZ) and Jasmonate Insensitive 1 (JIN1, also known as MYC2) (Kazan and Manners, 2008). JAR1 was targeted by sly‐miR827‐5p (Figures 10 and 11b) whose expression was decreased (−2.50‐fold) in sensitive upground tissues, while increased (1.02‐fold) in tolerant upground samples in response to dehydration stress (Figures 3 and 4c, Table S2). The root tissues were not affected by drought, but generally miR827‐5p expression was higher in roots (Figure 3, Table S2). In same signalling cascade, JAZ receptor was targeted by sly‐miR169a‐5p (Figures 10 and 11b). In our results, sly‐miR169a‐5p expression was decreased in all tissues with drought (Figure 4a, b, Table S2). miR169 targets Jasmonate ZIM Domain (JAZ) and nuclear transcription factor Y subunit A‐3 (NFYA‐3) in tomato fruits (Karlova *et al*., 2013) and this is further validated in our study (Table S3). Additionally, sly‐miR169 expression was increased in tomato by drought treatment, while three *Sl*NFYA (1/2/3) genes were down‐regulated (Zhang *et al*., 2011). Moreover, overexpression of sly‐miR169c caused significantly down‐regulation of tomato target genes and induced the increased drought tolerance of tomato (Zhang *et al*., 2011), whereas overexpression of miR169a led to drought sensitivity in *Arabidopsis* plants (Li *et al*., 2008b). Our results were similar with *Arabidopsis* result indicating NFYA up‐regulation by drought in an ABA‐dependent manner (Li *et al*., 2008b). ABA‐dependent signalling of drought results in stomatal closure (Figure 10), and stomatal closure is the first response of plants to drought stress (Schroeder *et al*., 2001). This response is controlled by not only ABA, but also interactions of ABA with the other phytohormones JA, ethylene, auxin and cytokinine (Nemhauser *et al*., 2006). ABA and JA positively regulate the stoma closure, while auxin and cytokinine regulate negatively and ethylene response depends on tissues and stresses (Daszkowska‐Golec and Szarejko, 2013; Huang *et al*., 2008; Nemhauser *et al*., 2006). Excitingly, 48 miRNAs target plant hormone signal transduction pathway (Figures 10 and 11b).

In conclusion, we identified 699 known miRNAs and the majority of them were expressed significantly in response to drought stress in a tissue‐ and genotype‐specific manner. According to the GO and KEGG analyses, the majority of these miRNAs involved in response to hormone stimulus/reactive oxygen species/water deprivation/salt stress, signal transduction, fruit, shoot, seed and root development (Figures 7a and 8) and plant–pathogen interaction, biosynthesis of plant hormones and plant hormone signal transduction pathways (Figures 9 and 10). Drought‐responsive miRNAs (such as sly‐miR160, sly‐miR165, sly‐miR166, sly‐miR171, sly‐miR398, sly‐miR408, sly‐miR827, sly‐miR9472, sly‐miR9476 and sly‐miR9552) regulated drought and development‐associated genes like DRP, HD‐ZIP, MYB, NAC and PSII in root and upground tissues (Figure 11a). Likewise, sly‐miR169, sly‐miR172, sly‐miR393, sly‐miR5641, sly‐miR5658 and sly‐miR7997 function in plant hormone signal transduction pathway and related proteins (Figures 10 and 11b). These results reveal drought‐responsive miRNA profiles of drought‐sensitive and drought‐tolerant tomato genotypes in tissue‐specific pattern and contribute to the development of drought‐tolerant tomato plants.

## Materials and methods

### Plant material and drought treatment

The seeds of drought‐sensitive (CGN24169: *Lycopersicon esculentum*, L.M.I‐56) and drought‐tolerant (CGN18399: *L. esculentum* var. *cerasiforme*, Tomatillo; PI 187002 selection 1) tomato genotypes were obtained from the Centre for Genetic Resources, The Netherlands (CGN), Wageningen University and Research Centre, The Netherlands. The seeds were surface‐sterilized with 75% (v/v) ethanol for 15 s, followed by 20% bleach (v/v) for 15 min and washed with sterilized distilled water for at least three times. Sterilized seeds were germinated on MS (Murashige and Skoog, 1962) medium (pH 5.8), containing 3% sucrose and 0.8% agar in a growth chamber with fluorescent light (~1400/mol^2^/ms) under 16‐h light /8‐h dark cycle at 25 ± 2 °C for 14 days. For drought treatment, 14‐day‐old seedlings were exposed to 5% polyethylene glycol for 7 days. For control and drought treatments, four seeds were germinated in Magenta boxes and the experiments were replicated six times in six individual vessels. After stress treatment for 7 days, the root and upground tissues were collected from seedlings and immediately frozen in liquid nitrogen. The samples were stored at −80 °C till RNA extraction.

### Small RNA library construction and sequencing

Total RNAs were extracted from root and upground tissues of drought‐sensitive and tolerant tomato plants using the *mir*Vana^™^ miRNA Isolation Kit (Ambion, Austin, TX) according to the manufacturer's instructions. The quality and quantity of RNAs were measured with a NanoDrop ND‐2000 spectrophotometer (Thermo Scientific, Wilmington, DE). RNA isolation was carried out individually for each sample with four biological replicates, then the RNAs were sent to BGI (Shenzen, China) for small RNA library construction and high‐throughput sequencing using Illumina HiSeq2000 platform.

### Identification of tomato miRNAs using deep sequencing

The raw reads were first cleaned up, including removing adapter sequences and eliminating low‐quality reads. Then, the length distribution of clean reads was categorized to analyse the composition of small RNA data, and 16‐ to 28‐nt‐length small RNAs were used for further analysis. High‐quality clean small RNA tags were mapped to tomato genome (ftp://ftp.solgenomics.net/tomato_genome/annotation/ITAG2.4_release/ITAG2.4_genomic.fasta) by SOAP (short oligonucleotide alignment program) to find out their expression and distribution on the genome (Li *et al*., 2008a). Then, the matched tags were aligned to NCBI GenBank (http://www.ncbi.nlm.nih.gov/genbank/) (Benson *et al*., 2015) and Rfam 11.0 (http://rfam.xfam.org/) (Burge *et al*., 2013) databases using BLASTall and BLASTn to determine rRNA, tRNA, snRNA and snoRNAs. Following this search, repeat‐containing RNAs, sense and antisense exon and intron sequences were detected, and fully matched all RNA types excluding miRNAs were gotten rid of. Then, for annotation of remaining sequences, conserved miRNAs were mapped to miRBase Release (v21 on June 26th, 2014) database (http://www.mirbase.org/ftp.shtml) (Kozomara and Griffiths‐Jones, 2014) and researched for *L. esculentum* miRNAs. The expression of miRNAs was calculated simultaneously by summing the count of tags overlapping at least 16 nt and/or two mismatches with aligned known miRNAs in database. DEGseq package was used for differential expression analysis of miRNAs, after normalizing raw read numbers with trimmed mean of M‐values (TMM) normalization method (Robinson and Oshlack, 2010; Wang *et al*., 2010). For normalization, firstly the normalization factors were calculated and then normalized read numbers were determined using following formula: [raw read counts/(library size × normalization factor) × 10^6^] (Bai *et al*., 2014). Fold changes were calculated with the formula [log_2_(normalized read numbers of group2/group1)]. Then, for the identification of significantly expressed miRNAs, the criteria were used as if (fold change ≥1 or ≤−1) and (*P* or *q*‐value <0.05) (Storey, 2003). To show differential expression profile among drought‐sensitive and drought‐tolerant tomato root and upgrounds, heatmap was constructed for the most abundant 130 conserved using Qlucore Omics Explorer 3.0 (Qlucore AB) (http://www.qlucore.com/).

### Target prediction, GO enrichment and KEGG pathway analysis

Significantly expressed miRNAs were used for target prediction using psRNATarget server (http://plantgrn.noble.org/psRNATarget/) with default parameters, including maximum expectation score (3.0), length for complementarity (17 bp) and range of central mismatch (10–11 nt) (Dai and Zhao, 2011). *Solanum lycopersicum* (tomato) cDNA library, version 2.4 (ftp://ftp.solgenomics.net/tomato_genome/annotation/ITAG2.4_release/ITAG2.4_cdna.fasta) was used to predict target genes, and for functional annotation and enrichment analysis of target genes, agriGO (GO Analysis Toolkit and Database for Agricultural Community) web‐based tool was used (Du *et al*., 2010). Firstly, the protein sequences of target genes (ftp://ftp.solgenomics.net/tomato_genome/annotation/ITAG2.4_release/ITAG2.4_proteins.fasta) were aligned against *Arabidopsis* protein sequences (ftp://ftp.arabidopsis.org/home/tair/Sequences/blast_datasets/TAIR10_blastsets/TAIR10_pep_20101214_updated) to find out the homologues. Then, the matched gene list was submitted to agriGO query list as TAIR10 locus ID, and GO classification was performed. The enriched GO terms of biological process, molecular function and cellular component categories were visualized with DAGs (directed acyclic hierarchical graph) and bar charts and pathway analyses were performed using KEGG (The Kyoto Encyclopedia of Gene and Genome) database (http://www.genome.jp/kegg/kegg1.html) using target gene IDs as queries (Kanehisa *et al*., 2014). The relationship between drought‐responsive miRNAs and their putative targets was visualized using Cytoscape network platform (Saito *et al*., 2012).

### Validation of miRNA expressions with qRT‐PCR analysis

Total RNAs of tolerant root tissues belonging to drought‐treated and control samples isolated for deep sequencing were used to validate miRNA expression results. Firstly, stem‐loop reverse transcription (RT) was carried out using TaqMan^®^ MicroRNA Reverse Transcription Kit (Applied Biosystems, Foster City, CA). A total of 15 μL RT reaction contained 1 mm dNTPs with dTTP, 1× RT buffer, 50U MultiScribe^™^ Reverse Transcriptase, 3.8U RNase inhibitor and 500 ng total RNAs and nuclease‐free water. Also 1.3 μm miRNA‐specific stem‐loop RT primers were used to generate single‐stranded cDNA for miRNAs (Table S6). The following temperature program was used to perform RT reaction as 30 min at 16 °C, 30 min at 42 °C, 5 min at 85 °C, and then holding at 4 °C. Before quantitative real‐time PCR (qRT‐PCR), the cDNAs were diluted in 100 μL DNase/RNase‐free water. Eight miRNAs (sly‐miR156a‐5p, sly‐miR169e‐3p, sly‐miR172a, sly‐miR393a‐5p, sly‐miR399a‐5p, sly‐miR408b‐5p, sly‐miR482d‐3p and sly‐miR9472‐5p) were randomly selected, and specific forward primers and universal reverse primer were designed to amplify the miRNAs (Table S6). qRT‐PCRs were carried out using 2× SensiFAST SYBR^®^ Hi‐ROX mix (Bioline, Taunton, MA) on a Applied Biosystems 7300 Real‐Time PCR System. Briefly, each 20 μL reactions contained 10 μL SensiFAST mix, 6 μL nuclease‐free water, 2 μL cDNA product and 2 μL primer mix. The reactions were performed with the following temperature program: 10 min at 95 °C for enzyme activation, 40 cycles of 15 s at 95 °C for denaturation and 60 s at 60 °C for annealing/extension, followed by a dissociation step for 1 cycle of 95 °C for 15 s, 60 °C for 60 s, 95 °C for 15 s and 60 °C for 15 s. Three technical replicates for each biological reactions and three biological replicates were performed for root tissues of tolerant genotype. Actin‐7 gene was used for normalization of qRT‐PCR data. The fold changes were calculated using 2^‐(ΔΔCt)^ values, and relative expressions were shown as log_2_ fold changes.

### Degradome library construction, sequencing and data analysis

For degradome sequencing, the RNAs were first pooled from all samples at a same amount. Firstly, polyadenylated RNAs were isolated with oligo‐d(T) bead extraction. Then, MmeI recognition site carrying 5′‐RNA adapter was ligated to 5′‐end that has mRNA fragments of miRNA‐induced cleavage. Afterwards, the fragments were converted to cDNA by reverse transcription and amplified by PCR (German *et al*., 2009). The PCR products of degradome library were sequenced on Illumina HiSeq2000 sequencing system, and the adapter sequences, low‐quality reads and N‐containing fragments were filtered from the raw reads. After preprocessing, sRNAs were eliminated by Genbank and Rfam 11.0 databases, and then KEGG (Kanehisa *et al*., 2014) and NR (nonredundant) (ftp://ftp.ncbi.nih.gov/blast/db/FASTA/nr.gz) databases were used for the annotation of cleaved target genes. Then, clean tags were matched to the tomato genome (ITAG2.4 Release cDNA library) by SOAP2.20 (Li *et al*., 2009) with allowing only two mismatches, and with the classification of clean tags, the sense strand of tomato cDNA library were used to predict miRNA cleavage sites using CleaveLand v3.0.1 (August 26, 2011) pipeline (Addo‐Quaye *et al*., 2009). The potential targets of miRNAs were analysed by PAREsnip software with *P*‐value <0.05 (Folkes *et al*., 2012), and T‐plot figures were drawn.

## Supporting information


**Table S1** Summary of small RNA sequencing data quality set.
**Table S2** Differentially expressed known miRNAs in root and upground tissues of drought‐sensitive and tolerant tomato genotypes.
**Table S3** Target prediction results of differentially expressed significant miRNAs.
**Table S4** Identified miRNA target cleavage sites using degradome sequencing data.
**Table S5** Enriched GO terms of drought‐responsive miRNA targets in root and upground tissues of tomato.
**Table S6** Specific qRT‐PCR primers for the expression validation of selected miRNAs.
